# Chemoradiotherapy with concurrent gemcitabine and cisplatin with or without sequential chemotherapy with gemcitabine/cisplatin *vs* chemoradiotherapy with concurrent 5-fluorouracil in patients with locally advanced pancreatic cancer – a multi-centre randomised phase II study

**DOI:** 10.1038/sj.bjc.6605420

**Published:** 2009-11-10

**Authors:** R Wilkowski, S Boeck, S Ostermaier, R Sauer, M Herbst, R Fietkau, M Flentje, S Miethe, H D Boettcher, T Scholten, C J Bruns, H G Rau, A Hinke, V Heinemann

**Affiliations:** 1 Department for Radiotherapy and Radiooncology, Klinik Bad Trissl, Oberaudorf, Germany; 2 Department of Internal Medicine III, Klinikum Grosshadern, Ludwig-Maximilians-University of Munich, Munich, Germany; 3 Department of Radiotherapy, University of Erlangen, Erlangen, Germany; 4 Department of Radiotherapy, University of Regensburg, Regensburg, Germany; 5 Department of Radiotherapy, University of Rostock, Rostock, Germany; 6 Department of Radiotherapy, University of Würzburg, Würzburg, Germany; 7 Department of Radiotherapy, University of Leipzig, Leipzig, Germany; 8 Department of Radiotherapy, University of Frankfurt, Frankfurt, Germany; 9 Department of Internal Medicine, Krankenhaus, Hagen, Germany; 10 Department of Surgery, Klinikum Grosshadern, Ludwig-Maximilians-University of Munich, Munich, Germany; 11 Department of Surgery, Klinikum Dachau, Dachau, Germany; 12 WiSP Research Institute, Langenfeld, Germany

**Keywords:** chemoradiotherapy, cisplatin, gemcitabine, pancreatic cancer

## Abstract

**Background:**

No standard treatment for locally advanced pancreatic cancer (LAPC) is defined.

**Patients and methods:**

Within a multi-centre, randomised phase II trial, 95 patients with LAPC were assigned to three different chemoradiotherapy (CRT) regimens: patients received conventionally fractionated radiotherapy of 50 Gy and were randomised to concurrent 5-fluorouracil (350 mg m^−2^ per day on each day of radiotherapy, RT-5-FU arm), concurrent gemcitabine (300 mg m^−2^), and cisplatin (30 mg m^−2^) on days 1, 8, 22, and 29 (RT-GC arm), or the same concurrent treatment followed by sequential full-dose gemcitabine (1000 mg m^−2^) and cisplatin (50 mg m^−2^) every 2 weeks (RT-GC+GC arm). Primary end point was the overall survival (OS) rate after 9 months.

**Results:**

The 9-month OS rate was 58% in the RT-5-FU arm, 52% in the RT-GC arm, and 45% in the RT-GC+GC arm. Corresponding median survival times were 9.6, 9.3, and 7.3 months (*P*=0.61) respectively. The intent-to-treat response rate was 19, 22, and 13% respectively. Median progression-free survival was estimated with 4.0, 5.6, and 6.0 months (*P*=0.21). Grade 3/4 haematological toxicities were more frequent in the two GC-containing arms, no grade 3/4 febrile neutropaenia was observed.

**Conclusion:**

None of the three CRT regimens tested met the investigators' definition for efficacy; the median OS was similar to those previously reported with gemcitabine alone in LAPC.

Pancreatic adenocarcinoma remains a disease with a dismal prognosis: in 2008, 37 680 estimated new cases were diagnosed in the United States, with a nearly identical rate of estimated deaths (34 290) from pancreatic cancer (Jemal *et al*, 2008). Since the introduction of the nucleoside analogue gemcitabine, several phase III trials have evaluated the role of a gemcitabine-containing combination treatment for advanced pancreatic cancer (Burris *et al*, 1997; Hochster *et al*, 2006). So far, only the combination of gemcitabine with the anti-EGFR tyrosine kinase inhibitor erlotinib provided a survival benefit compared to single-agent gemcitabine (Moore *et al*, 2007). Promising efficacy results were also obtained with cytotoxic combinations of gemcitabine in combination with capecitabine or a platinum analogue (Cunningham *et al*, 2005; Louvet *et al*, 2005; Heinemann *et al*, 2006, 2008; Herrmann *et al*, 2007).

Most of the palliative phase III trials that were conducted during the last decade included both patients with non-resectable locally advanced pancreatic cancer (LAPC) and metastatic pancreatic cancer. However, based on *post hoc* subgroup analyses it became evident that LAPC may be characterised by a different disease biology than metastatic pancreatic cancer: patients with LAPC showed a prolonged survival (about 10 months) and in several studies, they obtained no survival benefit from combination chemotherapy (compared to gemcitabine alone) (Louvet *et al*, 2005; Heinemann *et al*, 2006). Chemoradiotherapy (CRT) also has been investigated widely in patients with LAPC; McGinn *et al* (2001) were among the first to introduce gemcitabine in CRT protocols for LAPC treatment. Subsequently, several other groups also conducted phase I and II clinical trials to improve CRT protocols for LAPC by including newer cytotoxic agents such as gemcitabine, cisplatin, or oxaliplatin as concurrent radiosensitising agents in their radiotherapy regimens (Desai *et al*, 2007; Haddock *et al*, 2007; Hong *et al*, 2008; Small *et al*, 2008). More recently, the final results from the first randomised trial comparing systemic chemotherapy alone with CRT (followed by maintenance chemotherapy) in LAPC were reported by Chauffert *et al* (2008): overall survival (OS), the primary study end point, was shorter in the CRT arm (8.6 months) than in the gemcitabine chemotherapy arm (13 months, *P*=0.03; Chauffert *et al*, 2008). In contrast, the preliminary results of a phase III trial (E4201) comparing gemcitabine in combination with radiotherapy *vs* gemcitabine alone in LAPC suggested a survival benefit for patients in the radiotherapy arm (11 *vs* 9.2 months). However, this study with 74 patients was terminated prematurely due to low accrual (targeted sample size, 316), and significant grade 3/4 toxicities were observed in both the arms (Loehrer *et al*, 2008).

The purpose of this three-arm, randomised phase II trial was to exploratively compare three different CRT regimens in terms of efficacy and tolerance in the treatment of patients with LAPC. A 5-fluorouracil (5-FU)-based CRT protocol was selected as reference arm, whereas patients in the two other treatment arms received CRT with concurrent low-dose gemcitabine and cisplatin. These two cytostatic agents were selected on the basis that both are known to exert their effect as potent radiosensitisers in pancreatic cancer and both agents are also known to be effective in patients with metastatic disease. In one treatment arm, patients also received sequential full-dose chemotherapy with gemcitabine/cisplatin (in analogy to Heinemann *et al*, 2006) after completion of concurrent gemcitabine/cisplatin-based CRT to improve systemic disease control.

## Patients and methods

### Patient population

Male or female patients between 18 and 75 years of age with histologically confirmed, non-resectable pancreatic cancer (stages III and IVA) were eligible for this phase II study. Non-resectability criteria included at least one of the following CT findings: nodal involvement; retroperitoneal infiltration; infiltration of the arteria mesenterica superior, vena mesenterica superior, arteria hepatica, or portal vein. At least one bi-dimensionally measurable lesion had to be present. Exclusion criteria included distant metastasis and previous radiotherapy of the abdominal region. The following patients were also excluded: pregnant or lactating patients, women of childbearing potential who lacked a reliable contraceptive method, patients with poor performance status (KPS <70%), insufficient renal function (creatinine clearance <80 ml min^−1^), and active infections. Patients who participated in another experimental clinical trial within 6 weeks of the start of treatment were ineligible. Adequate bone marrow reserve was required: WBC, ⩾3.5 × 10^9^ l^−1^; platelet count, ⩾100 × 10^9^ l^−1^; haemoglobin, ⩾100 g l^−1^. The study was approved by the ethical committees of all participating German centres and each patient gave written informed consent before any study-specific procedure. This study was conducted according to the Declaration of Helsinki Principles.

### Study design and treatment

In this explorative phase II trial the patients were randomised in a 1 : 1 : 1 ratio to the three treatment arms, after stratification for performance status and centre. The primary objective of the study was to determine the anti-neoplastic efficacy of the combined modality regimens, primarily about the OS rates at 9 months after randomisation. This specific 9-month interval was selected as the study committee expected a minimum median survival time of 9 months for LAPC patients treated with CRT. Secondary objectives included the achievement of secondary resectability, progression-free survival (PFS), response rate, and toxicity.

External beam irradiation according to CT or MRT image-based three-dimensional planning was administered on 5 days per week at a daily dose of 2.0 Gy to the first-order target volume (primary tumour with a 1 cm margin) and of 1.8 Gy to the second-order target volume (regional lymph node (LN) areas with a 2–3 cm margin). Thus, the total planned dosages were 50 and 45 Gy respectively. In the reference arm, concurrent 5-FU was given as a 24 h continuous infusion of 350 mg m^−2^ per day on each day of radiotherapy (RT-5-FU arm). In the experimental CRT arm 300 mg m^−2^ gemcitabine and 30 mg m^−2^ cisplatin were administered i.v. on days 1, 8, 22, and 29 (ie on the first day of irradiation weeks 1, 2, 4, and 5), 1 h before start of radiotherapy (RT-GC arm). In the trial arm that included sequential systemic chemotherapy (RT-GC+GC arm), patients without disease progression after gemcitabine/cisplatin-based CRT (analogous to RT-GC) received gemcitabine (1000 mg m^−2^ over 30 min) and cisplatin (50 mg m^−2^) every 2 weeks until disease progression, resectability of the tumour, or unacceptable toxicity.

If necessary, protocol-defined dose reductions were performed according to clinical and laboratory parameters. Supportive treatment (e.g. anti-emetic therapy) was administered according to local standards of the participating centres.

### Efficacy and tolerance evaluation

Pre-treatment evaluation included complete history and physical examination, assessment of performance status and disease symptoms, a helical CT of the abdomen with assessment of the tumour size, and an MRI of the liver. Regularly performed laboratory tests included blood counts, creatinine, liver enzymes, total bilirubin, alkaline phosphatase, total protein, albumin, CEA, and CA19-9. A re-staging by CT scan was performed 10 weeks after the start of radiotherapy and every 3 months thereafter. Remission or progression of the tumour was defined according to standard WHO criteria. Objective responses had to be confirmed by a second CT performed at least 4 weeks after the initial finding suggesting tumour regression.

For the determination of PFS and OS, all patients were randomised according to the protocol inclusion and exclusion criteria were included (intent-to-treat analysis). Toxicity was classified according to the National Cancer Institute Common Toxicity Criteria (NCI-CTC), version 2.0. All non-haematological adverse events were assessed according to the NCI-CTC grading system in this trial. In addition, acute toxicity (haematological and non-haematological) during concurrent CRT was also assessed using standard RTOG toxicity criteria.

### Statistical analyses

Based on the available data for the efficacy of standard treatment, a survival rate of 60% after 9 months was expected after 5-FU-based CRT in the reference arm. Thus, the experimental regimens with gemcitabine and cisplatin to be tested in this trial would be considered to be not sufficiently active, if the 9-month survival rate was lower than 60%, but promising in case of a 9-month survival of ⩾80%. According to these assumptions and to achieve a power of 90% and a type I error of 0.05, an optimal two-stage design by Simon required a sample size of 19 evaluable patients per study arm in the first stage. In this original design, the survival of at least 13 out of these 19 patients would have allowed to enter stage two in the respective arm, then recruiting further cases up to 53 per arm. Otherwise, the arm was to be closed for futility. To account for possible dropouts, we strived for a total number of 60 patients in each arm. Descriptive statistical methods including confidence intervals were used throughout the analysis. *P*-values presented in this report are explorative in nature and result from two-sided hypothesis tests.

As a matter of fact, none of the treatment arms reached the goal described above after the first step. However, as no promising alternatives for the treatment of LAPC were available at that point of time, the study committee decided to recruit another 30 patients to allow for a more precise estimate of efficacy and tolerability.

## Results

### Patient characteristics

**Figure 1:**
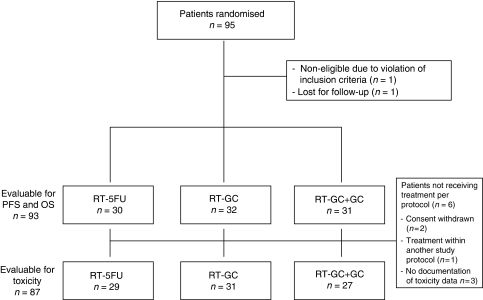
CONSORT diagram.

Between February 2002 and July 2005, 95 patients from 12 German centres were enrolled in this study; the database was closed for final analysis in August 2008. A CONSORT diagram of the trial is shown in Figure 1. One patient was classified as non-evaluable, as no study documentation was available, and one patient was lost to follow-up immediately after randomisation. Baseline patient characteristics are summarised in Table 1. Most patients had cT4 tumours, the highest rate of nodal involvement (N+) was observed in the RT-5-FU group (77%). However, in the RT-5-FU arm, 58% of randomised patients had a good performance status, whereas only 39% of patients in the RT-GC+GC arm had a KPS of 90–100%. Most of the pancreatic tumours were histologically classified as adenocarcinomas (about 85%); the remaining histologies were adenosquamous carcinoma and mucinous carcinoma. The most frequent reasons for non-resectability of the primary tumour in the pancreas were suspected LN involvement (peri-pancreatic LN, 39%; mesenterial LN, 23%; interaortocaval LN, 10%; liver hilus LN, 10%), vascular involvement (83%), and retroperitoneal involvement (13%).

### Treatment

#### Radiotherapy

The median duration of radiotherapy was 37 days (range, 5–98) in all patients (RT-5-FU, 37 days; RT-GC, 36 days; RT-GC+GC, 36 days). The median cumulative radiation dose to the first-order target volume was 50 Gy in each of the three treatment arms (range for all patients, 9–52). A dose reduction of radiotherapy was performed in 3% of the patients; in 5% of patients radiation treatment was delayed at least once during the course of the study.

#### Chemotherapy

Regarding the concurrent chemotherapy during radiation, gemcitabine dose reductions had to be performed in 19% of the patients in the RT-GC arm and in 39% of patients in the RT-GC+GC arm. The corresponding rates for cisplatin dose reductions were 19% (RT-GC) and 39% (RT-GC+GC) respectively. A dose reduction of concurrent 5-FU was necessary in 4% of the patients treated in the RT-5-FU arm. The main reasons for treatment postponement or dose reduction of concurrent chemotherapy were (based on 513 treatment cycles) organisational reasons (39 cycles, 8%), haematological toxicity (23 cycles, 4%), and non-treatment-related adverse events (16 cycles, 3%) respectively. Of the 31 patients randomised to RT-GC+GC, 6 (19%) received no sequential chemotherapy with gemcitabine and cisplatin; 6 (19%) received one cycle GC, 16 (52%) received two cycles, and 1 patient each (3%) three, four, and six cycles of GC.

The main reasons for termination of treatment were regular end of study therapy (44 patients, 47%), progressive disease (21 patients, 23%), death (12 patients, 13%), and wish of the patient (6%). In four patients (4%) study treatment was terminated because of an adverse event.

### Efficacy results

#### Response by imaging criteria and (secondary) resectability

Detailed results for objective response and resectability after CRT are summarised in Table 2. Overall, 70 patients were evaluable for objective response according to WHO criteria. Based on an intent-to-treat analysis, the overall response rate was 19% in the RT-5-FU arm, 22% in the RT-GC arm, and 13% in patients randomised to RT-GC+GC. Eighteen patients (19%) underwent a secondary operation after completion of study treatment; in eight of them, a R0 resection was achieved, four patients showed a microscopically positive resection margin (R1).

#### Survival results

**Figure 2:**
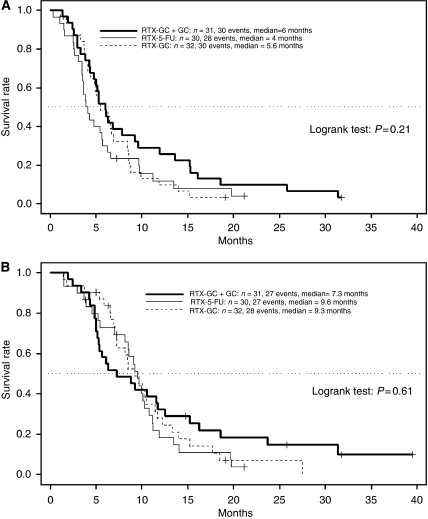
(**A**) Progression-free survival by treatment arm. (**B**) Overall survival by treatment arm. Abbreviations: 5-FU, 5-fluorouracil; C, cisplatin; G, gemcitabine; RTX, radiotherapy.

*Progression-free survival*: after a median follow-up of 8.6. months (range, 1.4–39.5), the median PFS was estimated with 4.0 months in the RT-5-FU arm, 5.6 months in the RT-GC arm, and 6.0 months in the RT-GC+GC arm (*P*=0.21; Table 3). The Kaplan–Meier plot for PFS is shown in Figure 2A.

*Overall survival*: at the time of final analysis, 82 of the 93 patients (88%) that were evaluated for OS had died. Median OS for patients randomised to RT-5-FU was 9.6 months, for patients randomised to RT-GC 9.3 months, and for patients randomly assigned to RT-GC+GC 7.3 months (Table 3). The corresponding 9-month OS rates were 58% (RT-5-FU), 52% (RT-GC), and 45% (RT-GC+GC) respectively (*P*=0.61). Figure 2B shows the Kaplan–Meier plot for OS.

### Tolerance results

#### RTOG acute toxicity during concurrent CRT

The acute toxicities during CRT (according to RTOG and NCI-CTC criteria) in each treatment arm are summarised in Table 4. Myelosuppression was more frequent with the two gemcitabine/cisplatin-containing CRT regimens, with leukocytopaenia and thrombocytopaenia being the most frequently observed haematological toxicity; no grade 3/4 febrile neutropaenia occurred. Classified according to RTOG criteria, grade 3/4 upper gastrointestinal (GI) tract toxicity was also more frequent in the two gemcitabine/cisplatin-based arms than in the RT-5-FU arm.

#### Non-haematological toxicity

A higher rate of nausea (all grades) was detected in the cisplatin-containing treatment arms (see Table 4). The rate of grade 3/4 infections (others than febrile neutropaenia) was low in all three arms, with 7% (RT-5-FU), 3% (RT-GC), and 0% (RT-GC+GC) respectively. In the study arm that contained a sequential chemotherapy with full-dose gemcitabine together with cisplatin (RT-GC+GC), the most frequent non-haematological grade 3/4 toxicities during maintenance chemotherapy were nausea (8%), vomiting (4%), fatigue (4%), and infection without neutropaenia (4%).

## Discussion

During the last years, LAPC has been increasingly recognised as a separate disease entity with specific biological features (Hochster *et al*, 2006). There is a scientific rationale to treat these patients with a combined modality approach: for example radiotherapy for local disease control and chemotherapy for distant disease control. The radiosensitiser 5-FU has been regarded as a standard agent for concurrent CRT in several GI malignancies, including pancreatic cancer, for several decades. Based on ‘chemotherapy only’ trials, a median survival of about 9–10 months can be expected for patients with LAPC that receive single-agent gemcitabine (Louvet *et al*, 2005; Heinemann *et al*, 2006). After gemcitabine was established as a standard of care in the palliative treatment of pancreatic cancer (Burris *et al*, 1997), several phase I/II trials were initiated to define the role of gemcitabine-based/-containing CRT for LAPC. Table 5 summarises selected phase II and III clinical trails (including the current study) that were conducted in LAPC and included gemcitabine and/or cisplatin in their treatment protocol. It becomes evident that various treatment approaches were investigated in these different trials: some studies applied concurrent low-dose gemcitabine and/or cisplatin as radiosensitisers, whereas others applied full-dose gemcitabine concurrently to radiotherapy. Although some studies also offered sequential full-dose chemotherapy after completion of CRT others did not. Thus the efficacy and tolerance of these different regimens can only be compared with several limitations.

This trial is the first to compare the efficacy and tolerance of three different CRT protocols in the treatment of LAPC: based on several efficacy end points (e.g. response rate, PFS, OS), RT-GC and RT-GC+GC were not distinctly superior compared with the reference arm RT-5-FU in this randomised phase II study. As expected, an increased haematological toxicity was observed in the two experimental arms, which was clinically manageable for both regimens (RT-GC and RT-GC+GC). Even if a comparison of survival results between different clinical trials is rejected as not appropriate, there is – at least to date – no clear scientific evidence to support the superiority of CRT compared with chemotherapy-only in LAPC. In this study, median OS times ranged between 7.3 months (RT-GC+GC) and 9.6 months (RT-5-FU). This observation is confirmed by the final results of a randomised trial that compared an intensive induction CRT (with concurrent 5-FU and cisplatin) followed by sequential full-dose gemcitabine with gemcitabine-only in LAPC: in this phase III study, patients in the CRT arm lived even shorter than patients treated with standard gemcitabine monochemotherapy (Chauffert *et al*, 2008). One explanation for this unexpected observation may be based on the fact that patients initially treated with CRT on that trial received significantly lower doses of sequential gemcitabine than patients in the single-agent gemcitabine arm (possibly associated with a significant haematological toxicity related to induction CRT).

Based on the data reported here, it remains unclear if gemcitabine can be regarded as a more effective radiosensitising agent compared to 5-FU in CRT regimens for LAPC. A previous randomised study by Li *et al* (2003) found early evidence for an improved time to progression and OS with the use of concurrent single-agent gemcitabine compared to 5-FU; however, this small study conducted in an Asian patient population used a different 5-FU bolus CRT regimen, and thus these results should only be compared carefully with the current trial. One limitation of this randomised phase II trial arises from the fact that an analysis of patterns of treatment failure (local *vs* distant) was not included. Specifically the prevention of local failure with the use of effective CRT should be regarded as an important palliative treatment goal in patients with LAPC.

Novel treatment concepts in LAPC are, for example, based on a ‘biological’ patient selection by systemic induction chemotherapy. Two study groups recently reported data on such an approach, where all LAPC patients receive initial systemic chemotherapy for 2–3 cycles and only patients with disease control carried on with CRT (Huguet *et al*, 2007; Krishnan *et al*, 2007). Prospective data from phase III trials that confirm a survival advantage for such a treatment regimen are still lacking; however, phase II data have shown the feasibility and tolerance of these concepts (Ko *et al*, 2007; Moureau-Zabotto *et al*, 2008). Moreover, novel innovative CRT regimens for LAPC including newer agents (e.g. oral fluoropyrimidines and biologicals) are currently under investigation in phase I and II clinical trials (Crane *et al*, 2006; Duffy *et al*, 2008; Kim *et al*, 2009; Michael *et al*, 2009).

In conclusion, none of the three CRT regimens investigated in this randomised phase II trial met the protocol pre-defined criteria for clinical efficacy (9-month OS rate >60%). The observed median OS times were similar to those previously reported with single-agent gemcitabine chemotherapy in LAPC. New treatment strategies are urgently required in patients with LAPC focusing on both an effective local disease control (eg with the use of innovative CRT regimens) and also a distant disease control with the use of effective systemic therapy.

## Figures and Tables

**Table 1:** Baseline patient and tumour characteristics (*n*=94)

	**RT-5-FU**	**RT-GC**	**RT-GC+GC**
**Characteristic**	**No. (%)**	**No. (%)**	**No. (%)**
Evaluable patients	31	32	31
			
*Age (years)*			
Median	63	63	65
Range	42–74	40–75	41–75
			
*Sex*			
Male	15 (48)	16 (50)	20 (65)
Female	16 (52)	16 (50)	11 (35)
			
*T stage*			
T2	1 (3)	—	—
T3	7 (23)	8 (25)	8 (26)
T4	23 (74)	24 (75)	23 (74)
			
*N stage*			
N0	5 (16)	12 (38)	12 (39)
N1	24 (77)	20 (62)	17 (55)
Nx	2 (6)	—	2 (6)
			
*Performance status*			
KPS 70–80%	12 (39)	15 (47)	17 (55)
KPS 90–100%	18 (58)	15 (47)	12 (39)
Missing	1 (3)	2 (6)	2 (6)
			
*Primary tumour site*			
Head	25 (81)	22 (69)	20 (65)
Body	6 (19)	6 (19)	6 (19)
Tail	—	1 (3)	2 (6)
Overlapping	—	3 (9)	3 (10)
Adenocarcinoma	27 (87)	27 (84)	27 (87)
			
*Histological degree of tumour differentiation*			
G1	2 (6)	3 (9)	1 (3)
G2	9 (29)	13 (41)	15 (48)
G3	13 (42)	11 (34)	11 (35)
Unknown	7 (23)	5 (16)	4 (13)

Abbreviations: 5-FU=5-fluorouracil; C=cisplatin; G=gemcitabine; KPS=Karnofsky performance status; RT=radiotherapy.

**Table 2:** Efficacy results: objective response rates by WHO criteria and secondary tumour resection (intention-to-treat analysis)

	**RT-5-FU (*n*=31)**	**RT-GC (*n*=32)**	**RT-GC+GC (*n*=31)**
	**No. (%)**	**No. (%)**	**No. (%)**
*Response*			
Complete response	0 (0)	2 (6)	0 (0)
Partial response	6 (19)	5 (16)	4 (13)
Stable disease	5 (16)	11 (34)	10 (32)
Disease control rate^a^	11 (35)	18 (56)	14 (45)
Progressive disease	12 (39)	9 (28)	6 (19)
Not assessable	8 (26)	5 (16)	11 (35)
			
*Secondary resection*			
Yes	4 (13)	8 (25)	6 (19)
No	27 (87)	24 (75)	25 (81)

Abbreviations: 5-FU=5-fluorouracil; C=cisplatin; G=gemcitabine; RT=radiotherapy.

a Disease control rate=rate of complete response+partial response+stable disease.

**Table 3:** Efficacy results: PFS and OS (intention-to-treat analysis)

	**RT-5-FU (*n*=30)**	**RT-GC (*n*=32)**	**RT-GC+GC (*n*=31)**
Median PFS (months)	4.0	5.6	6.0
95% CI	3.5–6.2	4.4–8.5	4.8–11.9
Median OS (months)	9.6	9.3	7.3
95% CI	8.5–11.1	7.3–12.2	5.3–15.2
9-month OS rate (%)	58	52	45
95% CI	43–80	37–74	31–67
18-month OS rate (%)	11	11	22
95% CI	4–32	4–31	11–43

Abbreviations: 5-FU=5-fluorouracil; C=cisplatin; CI=confidence interval; G=gemcitabine; OS=overall survival; PFS=progression-free survival; RT=radiotherapy.

**Table 4:** Toxicity results according to RTOG and NCI-CTC, version 2.0 (maximum per patient)

	**Percentage of patients**					
	**RT-5-FU (*n*=29)**		**RT-GC (*n*=31)**		**RT-GC+GC (*n*=27)**	
	**Grade**		**Grade**		**Grade**	
**Toxicity**	**1–2**	**3–4**	**1–2**	**3–4**	**1–2**	**3–4**
*RTOG acute toxicity*						
Leukocytopaenia	46	4	48	52	34	62
Thrombocytopaenia	18	4	31	52	46	38
Anaemia	54	0	79	7	77	4
Upper GI tract	39	0	28	20	42	8
Lower GI tract	15	4	10	10	12	0
Skin	7	0	10	0	4	0
						
*Non-haematological toxicity (NCI-CTC)*						
Fatigue	34	10	26	13	63	4
Weight loss	34	0	45	3	49	0
Diarrhoea	24	10	29	3	45	0
Nausea	62	0	74	13	89	4
Febrile neutropaenia	3	0	3	0	8	0
Infection without neutropaenia	24	7	29	3	26	0

Abbreviations: 5-FU=5-fluorouracil; C=cisplatin; G=gemcitabine; RT=radiotherapy.

**Table 5:** Efficacy results from selected phase II and III trials in LAPC

**Treatment protocol (Reference)**	**Phase**	**No. of patients**	**Regimen**	**PFS**/**TTP (months)**	**OS (months)**
RT/cGem (Small Jr *et al*, 2008)	II	41	RT: 36 Gy, cGem: 1000 mg m^−2^ (days 1, 8, 15)	NA	1y-OS rate: 73%
RT/cGem-Cis + sGem (Haddock *et al*, 2007)	II	48	RT: 50.4 Gy, cGem: 30 mg m^−2^, cCis: 10 mg m^−2^ (twice weekly)	7.3	10.2
RT/cGem-Cis (Hong *et al*, 2008)	II	41	RT: 45 Gy, cGem: 1000 mg m^−2^ (weekly)	8.9	16.7
			cCis: 70 mg m^−2^ (days 1, 29)		
RT/cGem + sGem *vs* Gem (Loehrer *et al*, 2008)	III	74	RT: 50.4 Gy, cGem: 600 mg m^−2^, sGem: 1000 mg m^−2^ (both weekly) *vs* Gem 1000 mg m^−2^ (weekly)	6.0 *vs* 6.7	11.0 *vs* 9.2^*^
RT/c5-FU-Cis + sGem *vs* Gem (Chauffert *et al*, 2008)	III	119	RT: 60 Gy, c5-FU: 300 mg m^−2^ per day, cCis: 20 mg m^−2^ per day	NA	8.6 *vs* 13.0^+^
			sGem: 1000 mg m^−2^ (weekly) *vs* Gem 1000 mg m^−2^ (weekly)		
RT/c5-FU *vs* RT/cGem-Cis *vs* RT/cGem-Cis + sGem-Cis (this study)	II	95	see Material and Methods	4.0 *vs* 5.6 *vs* 6.0	9.6 *vs* 9.3 *vs* 7.3

Abbreviations: 5-FU=5-fluorouracil; Cis=cisplatin; c=concurrent chemotherapy; CRT=chemoradiotherapy; Gem=gem; RT=radiotherapy; s=sequential chemotherapy.

^*^P=0.034; ^+^*P*=0.03.

## References

[bib1] Burris HA, Moore MJ, Andersen J, Green MR, Rothenberg ML, Modiano MR, Cripps MC, Portenoy RK, Storniolo AM, Tarassoff P, Nelson R, Dorr FA, Stephens CD, von Hoff DD (1997) Improvements in survival and clinical benefit with gemcitabine as first-line therapy for patients with advanced pancreas cancer: a randomized trial. J Clin Oncol 15: 2403–2413 9196156 10.1200/JCO.1997.15.6.2403

[bib2] Chauffert B, Mornex F, Bonnetain F, Rougier P, Mariette C, Bouché O, Bosset JF, Aparicio T, Mineur L, Azzedine A, Hammel P, Butel J, Stremsdoerfer N, Maingon P, Bedenne L (2008) Phase III trial comparing intensive induction chemoradiotherapy (60 Gy, infusional 5-FU and intermittent cisplatin) followed by maintenance gemcitabine with gemcitabine alone for locally advanced unresectable pancreatic cancer. Definitive results of the 2000–01 FFCD/SFRO study. Ann Oncol 19: 1592–1599 18467316 10.1093/annonc/mdn281

[bib3] Crane CH, Ellis LM, Abbruzzese JL, Amos C, Xiong HQ, Ho L, Evans DB, Tamm EP, Ng C, Pisters PW, Charnsangavej C, Delclos ME, O'Reilly M, Lee JE, Wolff RA (2006) Phase I trial evaluating the safety of bevacizumab with concurrent radiotherapy and capecitabine in locally advanced pancreatic cancer. J Clin Oncol 24: 1145–1151 16505434 10.1200/JCO.2005.03.6780

[bib4] Cunningham D, Chau I, Stocken D, Davies C, Dunn J, Valle J, Smith D, Steward W, Harper P, Neoptolemos J (2005) Phase III randomised comparison of gemcitabine (GEM) *vs* gemcitabine plus capecitabine (GEM-CAP) in patients with advanced pancreatic cancer. Eur J Cancer 3: 4 (suppl; abstr PS11)

[bib5] Desai SP, Ben-Josef E, Normolle DP, Francis IR, Greenson JK, Simeone DM, Chang AE, Colletti LM, Lawrence TS, Zalupski MM (2007) Phase I study of oxaliplatin, full-dose gemcitabine, and concurrent radiation therapy in pancreatic cancer. J Clin Oncol 25: 4587–4592 17925553 10.1200/JCO.2007.12.0592

[bib6] Duffy A, Kortmansky J, Schwartz GK, Capanu M, Puleio S, Minsky B, Saltz L, Kelsen DP, O'Reilly EM (2008) A phase I study of erlotinib in combination with gemcitabine and radiation in locally advanced, non-operable pancreatic adenocarcinoma. Ann Oncol 19: 86–91 17878176 10.1093/annonc/mdm441

[bib7] Haddock MG, Swaminathan R, Foster NR, Hauge MD, Martenson JA, Camoriano JK, Stella PJ, Tenglin RC, Scheafer PL, Moore Jr DF, Alberts SR (2007) Gemcitabine, cisplatin, and radiotherapy for patients with locally advanced pancreatic adenocarcinoma: results of the North Central Cancer Treatment Group phase II study N9942. J Clin Oncol 25: 2567–2572 17577035 10.1200/JCO.2006.10.2111

[bib8] Heinemann V, Quietzsch D, Gieseler F, Gonnermann M, Schoenekaes H, Rost A, Neuhaus H, Haag C, Clemens M, Heinrich B, Vehling-Kaiser U, Fuchs M, Fleckenstein D, Gesierich W, Uthgenannt D, Einsele H, Holstege A, Hinke A, Schalhorn A, Wilkowski R (2006) Randomized phase III trial of gemcitabine plus cisplatin compared with gemcitabine alone in advanced pancreatic cancer. J Clin Oncol 24: 3946–3952 16921047 10.1200/JCO.2005.05.1490

[bib9] Heinemann V, Boeck S, Hinke A, Labianca R, Louvet C (2008) Meta-analysis of randomized trials: evaluation of benefit from gemcitabine-based combination chemotherapy applied in advanced pancreatic cancer. BMC Cancer 8: 82 18373843 10.1186/1471-2407-8-82 PMC2292732

[bib10] Herrmann R, Bodoky G, Ruhstaller T, Glimelius B, Bajetta E, Schüller J, Saletti P, Bauer J, Figer A, Pestalozzi B, Köhne CH, Mingrone W, Stemmer SM, Tàmas K, Kornek GV, Koeberle D, Cina S, Bernhard J, Dietrich D, Scheithauer W (2007) Gemcitabine plus capecitabine compared with gemcitabine alone in advanced pancreatic cancer: a randomized, multicenter, phase III trial of the Swiss Group for Clinical Cancer Research and the Central European Cooperative Oncology Group. J Clin Oncol 25: 2212–2217 17538165 10.1200/JCO.2006.09.0886

[bib11] Hochster HS, Haller DG, de Gramont A, Berlin JD, Philip PA, Moore MJ, Ajani JA (2006) Consensus report of the International Society of Gastrointestinal Oncology on therapeutic progress in advanced pancreatic cancer. Cancer 107: 676–685 16847885 10.1002/cncr.22036

[bib12] Hong SP, Park JY, Jeon TJ, Bang S, Park SW, Chung JB, Park MS, Seong J, Lee WJ, Song SY (2008) Weekly full-dose gemcitabine and single-dose cisplatin with concurrent radiotherapy in patients with locally advanced pancreatic cancer. Br J Cancer 98: 881–887 18301403 10.1038/sj.bjc.6604247 PMC2266862

[bib13] Huguet F, André T, Hammel P, Artru P, Balosso J, Selle F, Deniaud-Alexandre E, Ruszniewski P, Touboul E, Labianca R, de Gramont A, Louvet C (2007) Impact of chemoradiotherapy after disease control with chemotherapy in locally advanced pancreatic adenocarcinoma in GERCOR phase II and III studies. J Clin Oncol 25: 326–331 17235048 10.1200/JCO.2006.07.5663

[bib14] Jemal A, Siegel R, Ward E, Hao Y, Xu J, Murray T, Thun MJ (2008) Cancer statistics, 2008. CA Cancer J Clin 58: 71–96 18287387 10.3322/CA.2007.0010

[bib15] Kim HM, Bang S, Park JY, Seong J, Song SY, Chung JB, Park SW (2009) Phase II trial of S-1 and concurrent radiotherapy in patients with locally advanced pancreatic cancer. Cancer Chemother Pharmacol 63: 535–541 18828020 10.1007/s00280-008-0836-1

[bib16] Ko AH, Quivey JM, Venook AP, Bergsland EK, Dito E, Schillinger B, Tempero MA (2007) A phase II study of fixed-dose rate gemcitabine and low-dose cisplatin followed by consolidative chemoradiation for locally advanced pancreatic cancer. Int J Radiat Oncol Biol Phys 68: 809–816 17363191 10.1016/j.ijrobp.2007.01.005

[bib17] Krishnan S, Rana V, Janjan NA, Varadhachary GR, Abbruzzese JL, Das P, Delclos ME, Gould MS, Evans DB, Wolff RA, Crane CH (2007) Induction chemotherapy selects patients with locally advanced, unresectable pancreatic cancer for optimal benefit from consolidative chemoradiation therapy. Cancer 110: 47–55 17538975 10.1002/cncr.22735

[bib18] Li CP, Chao Y, Chi KH, Chan WK, Teng HC, Lee RC, Chang FY, Lee SD, Yen SH (2003) Concurrent chemoradiotherapy treatment of locally advanced pancreatic cancer: gemcitabine *vs* 5-fluororuracil, a randomized controlled study. Int J Radiat Oncol Biol Phys 57: 98–104 12909221 10.1016/s0360-3016(03)00435-8

[bib19] Loehrer PJ, Powell ME, Cardenes HR, Wagner L, Brell JM, Ramanathan RK, Crane CH, Alberts SR, Benson AB (2008) A randomized phase III study of gemcitabine in combination with radiation therapy *vs* gemcitabine alone in patients with localized, unresectable pancreatic cancer: E4201. J Clin Oncol 26 (Suppl): (Abstr 4506)

[bib20] Louvet C, Labianca R, Hammel P, Lledo G, Zampino MG, André T, Zaniboni A, Ducreux M, Aitini E, Taieb J, Faroux R, Lepere C, de Gramont A (2005) Gemcitabine in combination with oxaliplatin compared with gemcitabine alone in locally advanced or metastatic pancreatic cancer: results of a GERCOR and GISCAD phase III trial. J Clin Oncol 23: 3509–3516 15908661 10.1200/JCO.2005.06.023

[bib21] McGinn CJ, Zalupski MM, Shureiqi I, Robertson JM, Eckhauser FE, Smith DC, Brown D, Hejna G, Strawderman M, Normolle S, Lawrence TS (2001) Phase I trial of radiation dose escalation with concurrent weekly full-dose gemcitabine in patients with advanced pancreatic cancer. J Clin Oncol 19: 4202–4208 11709563 10.1200/JCO.2001.19.22.4202

[bib22] Michael M, Price T, Ngan SY, Ganju V, Strickland AH, Muller A, Khamly K, Milner AD, Dilulio J, Matera A, Zalcberg JR, Leong T (2009) A phase I trial of capecitabine + gemcitabine with radical radiation for locally advanced pancreatic cancer. Br J Cancer 100: 37–43 19088724 10.1038/sj.bjc.6604827 PMC2634693

[bib23] Moore MJ, Goldstein D, Hamm J, Figer A, Hecht JR, Gallinger S, Au HJ, Murawa P, Walde D, Wolff RA, Campos D, Lim R, Ding K, Clark G, Voskoglou-Nomikos T, Ptasynski M, Parulekar W (2007) Erlotinib plus gemcitabine compared with gemcitabine alone in patients with advanced pancreatic cancer. A phase III trial of the National Cancer Institute of Canada Clinical trials group. J Clin Oncol 25: 1960–1966 17452677 10.1200/JCO.2006.07.9525

[bib24] Moureau-Zabotto L, Phélip JM, Afchain P, Mineur L, André T, Vendrely V, Lledo G, Dupuis O, Huguet F, Touboul E, Balosso J, Louvet C (2008) Concomitant administration of weekly oxaliplatin, fluorouracil continuous infusion, and radiotherapy after 2 months of gemcitabine and oxaliplatin induction in patients with locally advanced pancreatic cancer: A Groupe Coordinateur Multidisciplinaire en Oncologie phase II study. J Clin Oncol 26: 1080–1085 18309942 10.1200/JCO.2007.12.8223

[bib25] Small Jr W, Berlin J, Freedman GM, Lawrence T, Talamonti MS, Mulcahy MF, Chakravarthy AB, Konski AA, Zalupski MM, Philip PA, Kinsella TJ, Merchant NB, Hoffman JP, Benson AB, Nicol S, Xu RM, Gill JF, McGinn CJ (2008) Full-dose gemcitabine with concurrent radiation therapy in patients with nonmetastatic pancreatic cancer: a multicenter phase II trial. J Clin Oncol 26: 942–947 18281668 10.1200/JCO.2007.13.9014

